# Evolution of oxytocin pathways in the brain of vertebrates

**DOI:** 10.3389/fnbeh.2014.00031

**Published:** 2014-02-14

**Authors:** H. Sophie Knobloch, Valery Grinevich

**Affiliations:** Schaller Research Group on Neuropeptides, German Cancer Research Center (DKFZ), Max Planck Institute for Medical Research, University of HeidelbergHeidelberg, Germany

**Keywords:** oxytocin, hypothalamus, evolution, anatomy, release, behavior

## Abstract

The central oxytocin system transformed tremendously during the evolution, thereby adapting to the expanding properties of species. In more basal vertebrates (paraphyletic taxon *Anamnia*, which includes agnathans, fish and amphibians), magnocellular neurosecretory neurons producing homologs of oxytocin reside in the wall of the third ventricle of the hypothalamus composing a single hypothalamic structure, the preoptic nucleus. This nucleus further diverged in advanced vertebrates (monophyletic taxon *Amniota*, which includes reptiles, birds, and mammals) into the paraventricular and supraoptic nuclei with accessory nuclei (AN) between them. The individual magnocellular neurons underwent a process of transformation from primitive uni- or bipolar neurons into highly differentiated neurons. Due to these microanatomical and cytological changes, the ancient release modes of oxytocin into the cerebrospinal fluid were largely replaced by vascular release. However, the most fascinating feature of the progressive transformations of the oxytocin system has been the expansion of oxytocin axonal projections to forebrain regions. In the present review we provide a background on these evolutionary advancements. Furthermore, we draw attention to the non-synaptic axonal release in small and defined brain regions with the aim to clearly distinguish this way of oxytocin action from the classical synaptic transmission on one side and from dendritic release followed by a global diffusion on the other side. Finally, we will summarize the effects of oxytocin and its homologs on pro-social reproductive behaviors in representatives of the phylogenetic tree and will propose anatomically plausible pathways of oxytocin release contributing to these behaviors in basal vertebrates and amniots.

## Introduction

The concept of neurosecretion (Scharrer and Scharrer, 1945) was based on the discovery of large glandular cells (later named magnocellular neurons) that contained colloid product and resided in the hypothalamus of the teleost fish minnow *Phoxinus laevis* (Scharrer, 1928)^1^. A similar glandular cell type containing oxytocin (OT)- and vasopressin (VP)-like substances was—a few decades later—visualized by histochemical reactions (such as Gomori's method with aldehyde-fuchsin; Puchtler et al., 1979) in other vertebrates too. Indeed, the 60–80's of the 20th century were the time of extensive exploration of the phenomenon of neurosecretion (Scharrer, 1978), the diversity of nonapeptides (Acher, 1978) and the anatomy of hypothalamic neurosecretory centers (Polenov, 1978). One of the main directions at that time was the comparative anatomical analysis of hypothalamic nuclei in representatives of most vertebrate classes (Zeballos et al., 1967; Watkins, 1975; Moor and Lowry, 1998). Furthermore, the aspect of environmental physiology was excessively studied, focusing on migrating and spawning animals and monitoring challenges in the activity of their neurosecretory system during reproduction (Peter, 1977; Polenov et al., 1979; Arshavskaya et al., 1985). This direction of research led to fascinating environmental socio-biological insights into the contribution of hypothalamic neuropeptides on the formation of pair bonding in social mammalian and non-mammalian species (Carter et al., 1995; Goodson and Bass, 2000; Insel and Young, 2000; Goodson et al., 2009). However, the continuing shift toward studying the genetics, molecular biology and electrophysiology of the magnocellular neurons (Murphy et al., 2012) resulted in a deep understanding of detailed mechanisms but was lacking a general picture about the phylogenetic transformations of magnocellular neurons. We intend therefore to link the morphological transformations and the route of oxytocin release with the behavior observed in more basal vertebrates vs. amniots.

### Macroanatomical transformation of the hypothalamic-neurohypophysial system in vertebrates

**Figure 1:**
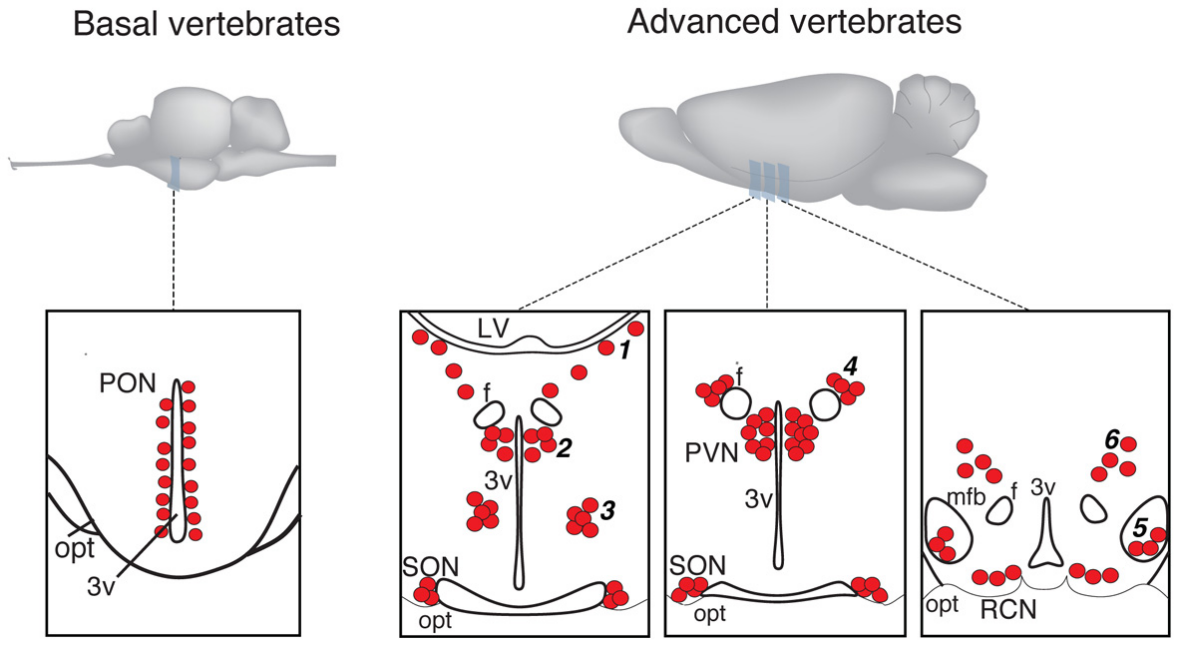
**Schematic presentation of magnocellular hypothalamic nuclei in representative examples of basal and advanced vertebrates (drawings are based on Grinevich and Polenov, 1994)**. 3v, third ventricle; F, columns of fornix; LV, lateral ventricle; MFB, medial forebrain bundle; OC, optic chiasm; OT, optic tract; PON, preoptic nucleus; PVN, paraventricular nucleus; SON, supraoptic nucleus. Accessory nuclei: 1—extrahypothalamic; 2—anterior commissural; 3—circular; 4—fornical; 5—nucleus of the medial forebrain bundle; 6—dorsolateral.

In more basal vertebrates (paraphyletic taxon *Anamnia*), composed by agnathans, fish and amphibians, magnocellular neurosecretory neurons express homologs of OT (mesotocin, isotocin, glumitocin, valitocin, aspargtocin) and VP (vasotocin) (Acher, 1978; Donaldson and Young, 2008). These neurons reside in the ancestral preoptic nucleus (PON; Diepen, 1962; Figure 1), which became recently a subject of genetic studies, using transgenic fish models (Gutnick et al., 2011; Herget et al., 2013). Magnocellular neurons of adult *Anamnia* are quite randomly distributed within the PON, existing intermingled with other types of cells. However, there is a ventro-dorsal gradient in size and morphology of neurons—while ventrally located neurons are rather small, more dorsally residing ones are bigger, and neurons reaching the upper pole of the PON are gigantic (Polenov, 1974; Garlov, 2005). This gradient reflects a “physiological regeneration” of the nucleus, which is caused by short periods of increased secretory activity (migration in fish and seasonal changes in frogs) and subsequent death of the gigantic neurosecretory neurons (Polenov, 1974; Garlov, 2005). This cell loss is, hence, compensated by newly born neurons (Chetverukhin and Polenov, 1993; Polenov and Chetverukhin, 1993). Although in non-mammalian species of vertebrates pronounced adult neurogenenesis is reported for various brain regions (see Kaslin et al., 2008 and Refs therein), in mammals this process is rather unique. Here it occurs only in specific areas, such as the subventricular zone and the dentate gyrus of the hippocampus (Ming and Song, 2011) as well as in the peptidergic hypothalamic arcuate nucleus, where cell turnover occurs at a low rate (Kokoeva et al., 2005).

In advanced vertebrates (monophyletic taxon *Amniota*: reptiles, birds, and mammals), there is a clear partition of magnocellular neurons in two separate nuclei—the paraventricular (PVN) and supraoptic (SON) nuclei (Meyer, 1935; Diepen, 1962; Figure 1). Some authors further subdivide the SON into main- and retrochiasmatic or postoptic part. However, the latter is absent in most evolutionarily conserved reptiles such as turtles (Fernández-Llebrez et al., 1988), and the retrochiasmatic part exists only in ancient mammals, such as platypus, that lack the typical SON. The PVN—in contrast to *Anamni*a's PON—is in rats composed by up to eight parts, and three of them comprise predominantly the magnocellular neurons (Swanson and Sawchenko, 1983; Armstrong, 2004; Simmons and Swanson, 2008). Although such strict territorial segregation is typical for rodents (especially for rats), but there are no reports on such segregations in other mammalian species, including humans (Swaab et al., 1993). Besides the main nuclei, PVN and SON, *Amniota* also possess groups of magnocellular neurons, termed accessory nuclei (AN)^2^, located in the territory between SON and PVN. There is some inconsistency in the naming of groups and their recognition as independent groups or parts of the PVN or SON. Some authors, for example, consider the “anterior commissural nucleus” (ACN) as an independent AN (Rhodes et al., 1981; Grinevich and Akmayev, 1997) while others classify it as division of the PVN (Swanson and Kuypers, 1980). Importantly, an AN of similar localization and composition (such as circular, fornical, and dorsolateral) exists in reptiles and various mammals (see Grinevich and Polenov, 1994). However, in birds—a highly specialized group of *Amniota*—the main and AN are not clearly bordered, and the subdivisions of PVN and SON as well as the AN are not homologous to those in other representatives of *Amniota* (Oksche and Farner, 1974; Grinevich and Polenov, 1994). Importantly, studies in rats (Rhodes et al., 1981) revealed that about 1/3 of all magnocellular neurons locate in AN, thereby pointing to their functional significance. In that line, we showed recently that the dorsolateral AN is the main source of OT projections to the central amygdala and is certainly involved in the attenuation of fear responses via OT release within this target structure (Knobloch et al., 2012).

The cause of the formation of a polycentric OT system in evolution is unclear. It could be speculated that the presence of the AN intermediate to the PVN and SON reflects the process of separation of the ancestral PON into the PVN and SON, leaving remnant cell groups in between. During this separation the dorsal part of the PON—the magnocellular preoptic nucleus—likely remained as PVN in amniotes as was recently shown in larval and adult zebra fish by comparing gene expression profiles with mammals (Herget et al., 2013). As for the SON, it was speculated that neurons located in the ventral PON migrate in ventro-lateral direction to the place of the later SON (Herget et al., 2013), leaving remaining cells of further AN. It is interesting that in one of the most primitive modern mammals—monotreme platypus *Ornithorhynchus anatinus*, most of the magnocellular OT neurons reside in the stream between the PVN and the retrochiasmastic part of the SON and never form the main nuclei found in other mammals (Ashwell et al., 2006).

The process of PON divergence in reptiles (paralleled by the first appearance of AN) coincides with the process of forebrain development (encephalization) and the respective formation of large fiber tracts connecting brainstem and spinal cord to the forebrain. The migrating magnocellular neurons and growing axonal bundles, such as the medial forebrain bundle could have been interfering with each other, as proposed in the following. During the embryogenesis of *Amniota*, magnocellular neurons possibly migrate along radial glia from the 3rd ventricle into ventro-lateral direction; the association of radial glia and magnocellular neurons was reported in the wallaby, the representative of marsupial mammals (Cheng et al., 2002). Similar migrations are known for the radial development of spinal cord, cerebellum and cortex (Hatten, 1999; Nadarajah and Parnavelas, 2002; McDermott et al., 2005) and are also observable in cell culture studies where neuroblasts migrate back and forth until finding their destination (Hatten, 1990). The bidirectional movement of magnocellular neurons might have been physically blocked by the growing fibers of the solid medial forebrain bundle (phylogenetically evolving in amphibians and reptiles; Herrick, 1910; Nieuwenhuys et al., 1982), thereby hindering neuronal migration from the supraoptic region back to the 3rd ventricle and entrapping cells (i.e., SON) latero-dorsally to the optic tract. This process of magnocellular nuclei formation in the embryogenesis (resembling phylogenetic development in accordance to Ernst Haeckel's law), in any case, requires further scientific investigations employing genetic and viral approaches combined with time-lapse imaging.

### Cytological changes in magnocellular neurons along the evolution

#### Dendro-ventricular contacts ^3^

**Figure 2:**
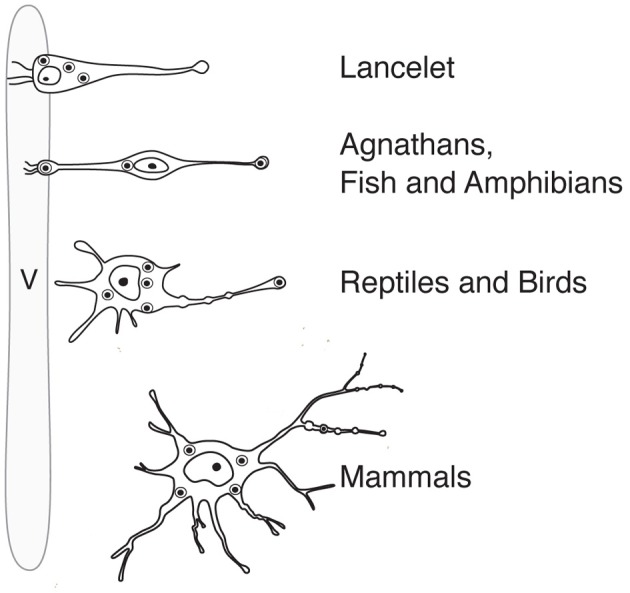
**Anatomy and position (in relation to the lumen of the ventricle) of magnocellular neurosecretory neurons in the hypothalamus of representatives of the phylogenetic tree**. v, ventricle.

**Figure 3:**
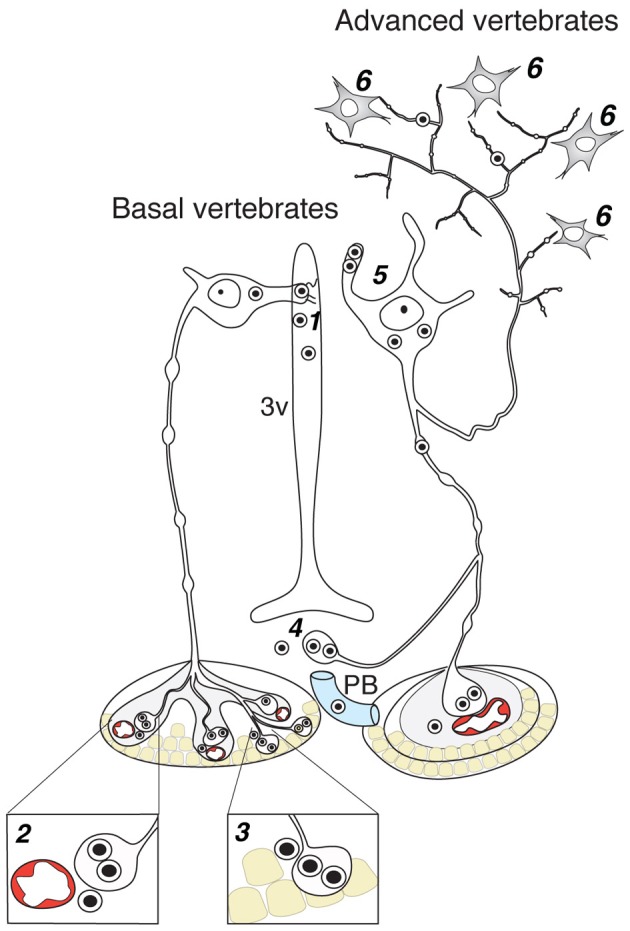
**Contacts of OT neurons and respective routes of OT release in the brain of basal and advanced vertebrates**. 1—dendro-ventricular contacts (trans-ventricular route of OT action); 2—axo-vasal contacts (release into systemic blood circulation); 3—axo-adenar contacts (paracrine action on adenotrophes); 4—axovasal contacts with portal venes; 5—dendritic release; 6—axonal release. 3v, third ventricle; PV, portal vessels.

**Figure 4:**
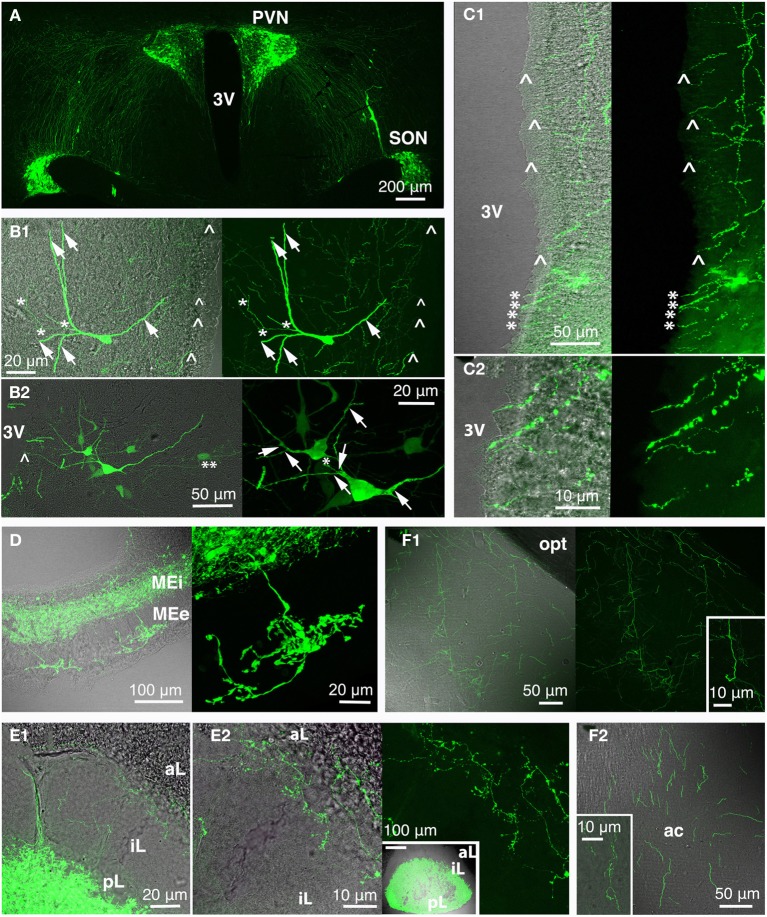
**Rat oxytocin (OT) neurons residing in PVN and SON (and AN; not depicted here) were visualized with the green fluorescence-protein Venus in rats using recombinant adeno-associated virus technique, thereby revealing the complexity of the central OT system (A) as well as of single OT neurons (B; arrow: dendrite/dendritic collateral; asterisk: likely axon/axonal collateral; double asterisk: contact to second OT cell; arrow head: fiber below ventricular ependyma)**. The 3rd ventricle is surrounded by OT fibers **(C)** that extend below the ventricle-lining ependymal layer (arrow head) or reach the ventricle lumen (asterisk; **C2** magnification of ventral part of the 3rd ventricle shown in **C1**). Release of OT into the circulation occurs via the median eminence (internal and external layer; **D**) and the posterior pituitary (**E**; **E2** inset shows a pituitary gland overview). Of note are OT fibers innervating the pituitary intermediate lobe in rats of reproductive state **(E)**. OT forebrain innervation for central OT release is likely the evolutionarily youngest features of the OT system **(F)**. Exemplarily, fibers in the medial amygdala **(F1)** and anterior olfactory nucleus **(F2)** are depicted (insets: magnified fibers) both structures functionally linked to reproductive and pro-social behaviors. 3V, 3rd ventricle; ac, anterior commissure; aL, anterior lobe; AN, accessory nuclei; iL, intermediate lobe; MEe, medial eminence, external layer; Mei, medial eminence, internal layer; opt, optic tract; pL, posterior lobe; PVN, paraventricular nucleus; SON, supraoptic nucleus.

Like probably many other neuronal cell types (Arendt, 2008), the hypothalamic magnocellular neurons underwent tremendous modifications in term of location and cytological organization during evolution (Polenov, 1978; Scharrer, 1978). The most primitive neurosecretory neurons were observed in *Amphioxus* (lancelet) (Obermüller-Wilén, 1979), which split from vertebrate ancestors ~550 million years ago (Gee, 2008; Figure 3). In *Amphioxus*, the neurosecretory cell bodies are lying between the ependymal cells and extend their axonal process through the inner wall of the ventricle to the ventral brain surface (Obermüller-Wilén, 1979). In fish, especially in the basal members of *Actinopterygii* (ray-finned fish) (e.g., sturgeon, sterlet), the cells extend their dendrites with expanded terminal parts into the lumen of the ventricle while their axons run away from the ventricle roughly at 90° angle. In addition, it seems that in *Anamnia* these dendrites are not only capable to release neuropeptides into the lumen of the third ventricle but also may sense (at least in the case of vasotocin neurons) via cilia the chemical content of the cerebro-spinal fluid (CSF, Tessmar-Raible et al., 2007). In mammals, a portion of these ventricle contacts seem to remain: using viral based technique the location of OT fibers (axons and/or dendrites) could be shown in intimate proximity to the 3rd ventricle and even in between of ependymal cells, contacting directly with the CSF (Figure 4C). Further along the phylogenic tree (see Figure 2) the majority dendrites and cell bodies of magnocellular neurons move away from the 3rd ventricle and undergo “neuronalization”^4^ forming rich dendritic trees and unique axonal specializations (the latter is described in great details in sections below). In respect of progressive changes of dendritic trees in evolution, it should be mentioned here that even in mammals (rats, dogs and monkeys) a fraction of OT neurons carries features of relatively simply organized neurons (Hatton, 1990; Armstrong, 1995; and references therein). These cells, visualized by Golgi (silver impregnation) technique, mostly reside in the SON, representing about half of neuronal population in this nucleus. They are bipolar neurons, similar to those observed in basal vertebrates, fish and frogs, while another half of SON neurons are multipolar cells with elaborated dendritic trees (Hatton, 1990; Armstrong, 1995, 2004; and references therein). The number of spines (as well as synapses) on dendrites of OT neurons is relatively modest (~500–600 synapses per OT neuron; William Armstrong, personal communication) especially compared to principle neurons of hippocampus (~10,000 synapses per single CA1 or CA3 neuron; Megias et al., 2001; Hosseini-Sharifabad and Nyengaard, 2007). However, during maternity period OT neurons undergo plastic changes (swelling, arborization) with ultrastructural reorganization of synaptic contacts (Stern and Armstrong, 1998; Theodosis and Poulain, 2001).

#### Axo-adenar contacts

Axo-adenar contacts are typical for magnocellular cells in *Anamnia*. Cells are sending axonal terminals close to the adenohypophysis (syn.: anterior pituitary, see Figure 3) where they are forming a root-like structure directly contacting adenohypophyseal cells. Hence, released neuropeptides affect subsequently the release of various pituitary hormones in paracrine fashion (Denef, 2008). However, the density of such contacts is gradually decreased in evolution. In fact, although we were able to detect a few examples of such contacts in adult rats (see Figure 4), only one paper reports on their presence in amniots: i.e., in the adenohypophysis of fetal sheep (Hoffman et al., 1989). The paracrine action of OT on pituitary cells (Hoffman et al., 1989) may occur during mammalian embryogenesis in the immature portal blood system. In general, the regress of direct axo-adenar contacts during evolution may parallel the process of anatomical separation of adenohypophysis and neurohypophysis by septal connective tissue (Enemar, 1960) and coincides with the development of an effective portal blood system from reptiles onwards (Enemar, 1960). By exception, in some highly specialized teleost fish (Baskaran and Sathyanesan, 1992) and advanced groups of amphibians, like anurans (Cruz, 1956; Lametschwandtner and Simonsberger, 1975), a portal system, albeit a primitive version, may already exist. Via the portal route, OT reaches epithelial cells of the adenohypophysis and modulates the release of adenotrophic hormones (Horn et al., 1985; Sheward et al., 1990; Denef, 2008).

#### Axo-vasal contacts

Endocrine neurosecretion in its classical meaning refers to the release of OT, VP and their homologs into the blood stream (Figure 3), which carries it to peripheral target organs such as the uterus, penis, mammary glands (also organs of the reproductive tract of non-mammalian species), the heart and also the skin (van Kesteren et al., 1995; Satake et al., 1999; Melis and Argiolas, 2011; Garrison et al., 2012; Gutkowska and Jankowski, 2012; Deing et al., 2013). Axo-vasal contacts are axonal terminals within the posterior pituitary lying in close proximity to fenestrated capillaries separated only by a basal membrane and the processes of pituicytes. This general structure of the posterior pituitary remained constant throughout the vertebrate evolution (Belenky, 1998) down to the phylogenetically old *Actinopterygii* (ray-finned) fish (Egorova et al., 2003). Besides this specialized structure, OT neurons also form axonal contacts with primary capillaries of the external zone of the median eminence (Figure 4). Hereby, OT is reaching the portal blood and directly acts on corticotrophes, lactotrophes, gonadotrophes and other cell types (Horn et al., 1985; Sheward et al., 1990; Denef, 2008).

Likely due to the vital importance of peripheral OT- and VP (and their homologs) hormones for reproductive physiology and water metabolism, the neurohypophysis exhibits a unique capacity for regeneration. After axonal damage of magnocellular neurons by pituitary stalk transection, the pituitary stalk undergoes an extensive hypertrophy and transforms into a new neurohemal organ, called “miniature neurohypophysis” (Spatz, 1958). This capacity of regeneration together with the astonishing survival of the magnocellular somata after axonal transection was demonstrated in both mammalian (including monkeys) and non-mammalian species (Atunes et al., 1979; Polenov et al., 1981; 1997).

### Routes of oxytocin release within the brain

As emphasized above, the evolutionarily oldest preserved OT processes contact the ventricle system (Figures 2, 4C). But given their rather low rate in mammals, the high OT concentrations in the CSF—exceeding those in blood (Kagerbauer et al., 2013)—likely arises from another source. Due to the fact that the CFS is composed of 1/3 extracellular fluid and 2/3 of blood plasma, the extracellular fluid, enriched by OT released from somas and dendrites of OT neurons (Ludwig and Leng, 2006) is most probably the main source of OT in the CSF (Landgraf and Neumann, 2004).

From an evolutionary point of view it is remarkable that OT homologs are present in primitive invertebrates species (such as annelids, nematods, mollusks, insects; van Kesteren et al., 1995; Satake et al., 1999; Stafflinger et al., 2008; Garrison et al., 2012), although no pituitary or other typical neuropeptide pathway through the body is available. Hence, the functional significance of evolving diverse distribution modes is not clear. However, it has been postulated that neuropeptides may initially have served as primitive neurotransmitters or modulators of neurotransmission (Jackson, 1980)—a functional implication that is still an aspect in mammalian species. Importantly, about 80% of the brain regions surrounding the ventricle system and the subarachnoid space express OT receptors in mammals. Therefore, diffusion of OT within the fluid of extracellular space (at least to a certain spatial extent) could be underlying behavioral effects of this neuropeptide in mammals, as found in countless studies with intracerebroventricular administration of OT during the last 30 years (Veening et al., 2010). It is assumed that intranasally applied in pharmacological doses (which are ~1000 times higher than the OT blood concentration; Huang et al., 2013; Neumann et al., 2013) OT may reach the CSF and exert substantially delayed and long lasting effects (starting from 30 to 45 min after application and lasting ~60–90 min) as was shown for various neuropeptides by Born et al. (2002). However, due to the short half-life of about 20 min of brain OT (Mens et al., 1983) it is unlikely that somatodendritically released OT reaches distant extrahypothalamic regions within a narrow time frame to achieve defined and rapid behavioral responses.

Simple uni- and bipolar cells forming ventricular contacts have been replaced during evolution by cells with extended dendritic trees (see Figure 2). This shift might have facilitated and intensified somatodendritic release of OT (Pow and Morris, 1989; Ludwig and Leng, 2006), which allows auto- and paracrine action of OT within OT-ergic nuclei under specific demand such as lactation (Landgraf and Neumann, 2004). Dendritically released OT is stimulating coordinated OT neuron activity during lactation, resulting in a pulsatile bolus release of OT into the blood (Lincoln et al., 1973). In parallel, OT release might be induced from axons in extrahypothalamic regions. This assumption was confirmed experimentally with 30 Hz optical stimulation, resembling the bursting activity of OT neurons during suckling (Wakerley and Lincoln, 1973; Poulain and Wakerley, 1982) and inducing axonal OT release in various brain regions (Knobloch et al., 2012, 2014)^5^.

There is a general agreement that parvocellular OT neurons project extensively toward the brainstem and spinal cord to form synaptic contacts with local neurons (Swanson and Sawchenko, 1983). However, these neurons are distinct from magnocellular ones in that they are not releasing OT into the systemic blood circulation. Although the presence of parvocellular OT-like neurons within the PON of *Anamnia*, e.g., teleost fish, was sporadically reported (Goodson et al., 2003; Thompson and Walton, 2013) the evolutionary transformation of this cell lineage has not been studied. Therefore, we leave this subject for further analysis, which will require the identification of genetic markers to specifically target parvocellular OT neurons.

During the pioneer times of neuroendocrine pathway research, ascending OT-ergic fibers were found in a limited number of extrahypohalamic forebrain regions such as the amygdala, bed nucleus of stria terminalis (BNST) and septal nuclei of rats (Buijs, 1978; Sofroniew, 1980), non-human primates (Atunes and Zimmerman, 1978; Kawata and Sano, 1982; Caffé et al., 1989; Wang et al., 1997) and human (Fliers et al., 1986) in addition to prominent descending brain stem- and spinal cord-innervating fiber tract. However, these studies suffered from technical limitations (such as deficient immunohistochemical feasibility) so that ascending fibers could only be revealed to a minor extent (Buijs, 1978; Sofroniew, 1980; Fliers et al., 1986). However, recent reports from Larry J. Young's (Ross et al., 2009) and our group (Knobloch et al., 2012), employing fluorogold- and viral-vector based techniques, respectively, clearly demonstrated that magnocellular OT neurons extensively innervate major forebrain regions in voles and rats. Interestingly, the number of OT axons in most forebrain regions is rather limited (Knobloch et al., 2012), hence explaining that they had been overlooked. The enormous number of OT molecules per large dense core vesicle (~85,000; Leng and Ludwig, 2008) and the extremely high (nM range) OT receptor affinity (Akerlund et al., 1999) still allows OT to sufficiently exert its effects in various forebrain regions. In line with this assumption, we demonstrated the functionality of sparse OT fibers *in vitro* and *in vivo*, as we showed that OT is released focally within the structure of demand, e.g., the lateral division of the central amygdala, and, hence, is capable to modify both microcircuit activity and amygdala-dependent behavior, namely conditioned fear response (Knobloch et al., 2012).

Interestingly, the focal, axonal OT release is, in spite of its spatial precision, not defined to a direct (synaptic) cell communication—a finding which is consonant with the initial idea of the Scharrers, who believed that the neurosecretory colloid can be released along the axon into the peri-axonal space (Scharrer, 1936; cited from Watts, 2011). Our hypothesis that OT acts non-synaptically is based on the fact that the onset of both electrophysiological and behavioral responses occur delayed, thereby exceeding the time typically needed for synaptic transmission (1–10 ms) and ranging within seconds in the central amygdala (Knobloch et al., 2012, 2014) and other brain regions, for example, in the anterior olfactory nucleus (personal communication from Dr. Wolfgang Kelsch, Central Institute of Mental Health and Heidelberg University). Importantly, a similar second-range delay of cellular responses was recently demonstrated after evoked somatodendritic release of VP from magnocellular PVN neurons, pointing on a similar non-synaptic, diffusion-like neuropeptide pathway that allows for interpopulational crosstalk within about 100 μm distance (Son et al., 2013). Besides the kinetics, the spatial distribution of large dense core vesicles, containing OT, also point on a non-synaptic transmission. The vesicles are not located in the active zones of pre-synapses in the few OT synapses found in the SON (Theodosis, 1985; Knobloch et al., in preparation) and ventromedial hypothalamic nucleus (Griffin et al., 2010) and OT receptors could not be attributed to the postsynaptic membrane. Taking all these arguments in account we propose that OT release from axons of magnocellular neurons in forebrain regions occurs by non-synaptic fashion. However, this should be further confirmed by the time-lapse imaging, implementing recently developed techniques for monitoring, docking and release of large dense core vesicles (de Wit et al., 2009; van de Bospoort et al., 2012). These techniques will also allow to dissect the role of glutamate-containing synaptic vesicles in OT neurons (Hrabovszky et al., 2006; Kawasaki et al., 2006), which remain enigmatic as no one was able to show fast synaptic transmission from axons of magnocellular OT neurons either in the hypothalamus or extrahypothalamic places (Knobloch et al., 2012, 2014).

Axonal projections to diverse brain areas are likely provided by distinct subgroups of OT neurons, implying an anatomical heterogeneity of OT neurons (Knobloch and Grinevich, personal observation). It is remarkable that there have been few if any studies on collaterals of OT neurons to different areas. Despite this, our ongoing research (manuscript in preparation) allows us to assume that in certain situations of life, such as love or fear, distinct populations of OT neurons may be activated, which—via specialized axonal projections—modulate specific brain areas and ultimately distinct behaviors in a pro-social or in-group supporting way. Indeed, recently we could show that associative fear learning induces the activation of a small subset of OT neurons, which specifically project to the central nucleus of the amygdala and, furthermore, evoked OT release from their axons within the central nucleus of amygdala readily attenuates fear response (Hasan et al., 2013; Kernert et al., 2013).

With respect to the evolution, there is a unique observation in a representative of the highly specialized and diverse group of teleost fish: in trout several mesotocin (and vasotocin) neurons project toward the forebrain (Saito et al., 2004). In analogy to rats (Knobloch et al., 2012), the authors furthermore demonstrated, using *in vitro* electrophysiology combined with biocytin-filling of cells, that magnocellular neurons of trout project to the posterior pituitary and—at the same time—to telencephalon and thalamus (Saito et al., 2004). This unique feature can be seen as an evolutionarily early advancement that later re-appeared in amniots. Indeed, ascending mesotocin or OT projections have been clearly demonstrated only in reptiles (Thepen et al., 1987; Silveira et al., 2002) and different mammals (Sofroniew, 1980; Fliers et al., 1986; Ross et al., 2009; Knobloch et al., 2012).

### Effects of OT and its homologs on pro-social and reproductive behavior of basal and advanced vertebrates

**Figure 5:**
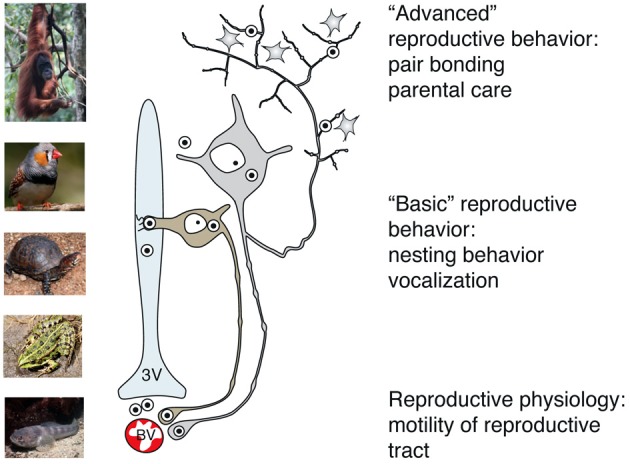
**Main pathways of OT release reflect its peripheral and central effects on reproduction in basal and advanced vertebrates**. While OT release into systemic blood circulation (via axo-vasal contacts) modulates motility of the reproductive tract, central OT release either into the cerebro-spinal fluid (via dendro-ventricular contacts) or into brain tissue (via axonal release) orchestrates reproductive behavior. Peripheral release of OT into the blood occurs in all vertebrates, however, release into the cerebro-spinal fluid is prevailing in basal vertebrates, which exhibit rather simple, stereotyped forms of OT-dependent reproductive behavior. In contrast, axonal OT release seemed to appear only in advanced vertebrates, especially in mammals. Taking in consideration the parallelism of appearance of OT axons in the forebrain and complex OT-mediated forms of reproductive and pro-social behavior, we speculate about a causative relation between these two processes in high vertebrates. 3v, 3rd ventricle; BV, blood vessels.

Since the turn of the last century the extract of the posterior pituitary has been known to stimulate contractions of the uterus and mammary glands (Oliver and Schäfer, 1895; Dale, 1909; Ott and Scott, 1910; Schäfer and Mackenzie, 1911). Subsequent comparative studies between numerous species conducted in the first half of the 20th century revealed that in both mammalian and non-mammalian species OT/mesotocin stimulates the activity of smooth muscle in reproductive tracts (Figure 5), furthermore the egg laying, sperm movement, ejaculation, as well as uterus contraction and milk let down in placental and non-placental mammals (Moore, 1992; Sebastian et al., 1998). Importantly, in non-placental marsupials OT and its homolog mesotocin co-exist in the hypothalamus. Together, they stimulate long lasting milk ejection (Nicholas, 1988), thereby prevailing different phases of the milk secretion to regulate lactation from neighboring breasts asynchronously, which is necessary for the contemporaneous development of offspring of different age (Nicholas, 1988; Sebastian et al., 1998).

Beside these neuroendocrine effects, countless publications convincingly demonstrate that in mammals OT is a key peptide for orchestrating reproductive, pro-social and in-group supporting behavior (Bosch and Neumann, 2012; Lukas and Neumann, 2013 and references therein). Based on that, OT is considered as a positive factor for species propagation (Lee et al., 2009) in all vertebrates. We here give a brief overview on aspects of OT involvement without providing a comprehensive analysis but rather a correlative view on the central OT pathways and the corresponding non-apeptide-mediated behaviors in vertebrates.

In a specialized marine teleost fish, the plainfin midshipman fish, Goodson and colleagues showed that central isotocin and vasotocin modulate social vocalization, in a sex- and type-specific manner (Goodson and Bass, 2000). Isotocin applied to the preoptic area of the anterior hypothalamus (the primary regions for endocrine and behavioral integration, e.g., in vocal production) modulates reproduction-unrelated social vocalization in females and type I males, both of which typically do not display parental care. In contrast, vasotocin applied to type II males, which are parental, modulates social vocalization according to the reproductive context—a courtship situation or the defense of the nest, eggs and hatchlings. Furthermore, isotocinergic axons were found in the ventral telencephalon and numerous hypothalamic and brainstem regions, which are components of ascending auditory pathways (Goodson et al., 2003). Unfortunately, there are no studies on the contribution of OT homologs to reproductive behaviors in agnathans, such as hagfish and lampreys, in cartilaginous fish (e.g., sharks and rays) or in primitive actinopterygians (e.g., sturgeon, beluga etc.). This gap makes it impossible to draw any definite conclusion about behavioral role of isotocin and its homologs in the first steps of vertebrate evolution.

In amphibians, especially in the evolutionarily advanced *Anura*, receptors for mesotocin are spread over brain regions implicated in reproductive behavior (Do-Rego et al., 2006). In addition, mesotocin is thought to stimulate the synthesis of neurosteroids, which target brain circuits controlling male calling and, again, reproductive behaviors (Do-Rego et al., 2006). Since in fish (except teleost) and amphibians isotocin/mesotocin projections reaching extrahypothalamic or reproduction-related brain regions could not be demonstrated, it is likely that these nonapeptides act trans-ventricularly, especially since courtship and reproductive behaviors do not require immediate effects and may last several days or weeks, depending on the species.

In reptiles, reports on OT effects are limited to nesting behavior (Carr et al., 2008). As in other nesting animals, typical nesting behavior in turtles consists of a sequence of actions such as nest-site selection, nest-site preparation, egg-cavity construction, oviposition and nest covering (Carr et al., 2008 and refs therein). Surprisingly, systemic application of OT (intramuscular injection) led to an atypical behavior with decoupled oviposition and nesting behavior, a phenomenon termed “false nesting” (Tucker et al., 1995). In turtles OT application evokes nest-covering behavior that precedes oviposition for up to 417 h (Carr et al., 2008). This study demonstrates that OT is powerful enough to induce nesting behavior even without egg laying. Involved central OT targets have yet not been dissected yet, and our literature search revealed only limited report on OT effects in reptilian reproductive behavior. However, further inside to this uniquely located group of animals—situated between basal vertebrates and mammals—would indisputably be beneficial for our understanding of the evolutionary role of OT homologs on the formation of behaviors as reptiles being the first group that carry a polycentric OT system with advanced multipolar neurons projecting extrahypothalamically. Presumably due to these achievements, reptiles display an extreme divergency of sexual behaviors, ranging from monogamous to “harem” behaviors (Bull, 2000; Godwin and Crews, 2002).

In birds, as shown in zebra finches, mesotocin seems to be a key peptide for the prolongation of time spent in large groups and—most importantly—with familiar conspecifics (Goodson et al., 2009). Furthermore, pro-social behavior elicited by central mesotocin infusion was dependent on the mesotocin receptor density in the lateral septum of female birds (Goodson et al., 2009). In fact, the reported effects of mesotocin resemble effects of OT on pair bonding observed in voles (Carter et al., 1995; Insel and Young, 2000). As in mammals with their specific OT fiber pattern, it is likely that also mesotocin-expressing species possess long-range axons to respective brain regions, such as to the lateral septum in birds, and regulate behavior with spatial precision.

In non-mammalian vertebrates vasotocin and its homologs modulate reproductive behavior and, in fact, seem to hold an even more important role than OT-like neuropeptides. Vasotocin is involved in the induction of vocalization, courtship behavior (like male amplectic clasping behavior), female sexual receptivity, alternative mating and many more social behaviors (Moore, 1983; Wilczynski et al., 2005; Balment et al., 2006; Soares et al., 2012). Such diverse effects in non-mammalians are not surprising since many extrahypothalamic vasotocin-expressing regions and the arising wide-spread projections are comparable to the extrahypothalamic VP system of mammals (de Vries and Miller, 1998). Summing up the impact of both peptides—OT/OT homologs and VP/VP homologs—in different species, it seems that the latter holds a dominant role in regulating reproductive behavior in fish and amphibians, while OT-like peptides are more important in birds (Goodson et al., 2012) and mammals (Lee et al., 2009), which display more complicated reproductive rituals. Nevertheless, the picture seems to be very complex as in many behavioral and cognitive aspects both peptides modulatory interact (Neumann, 2009; Bosch and Neumann, 2012; Stoop, 2012) and furthermore, as constituting a sexual dimorphic systems, vary in their relative priority in males or females (Veenema et al., 2013).

## Conclusions

During evolution OT-like genes and peptides remained highly conserved, which could be demonstrated via genomic integration of the OT homolog isotocin of the teleost *Fugu rubripes* (blowfish) in rat (Venkatesh et al., 1997; Murphy et al., 1998) and mouse (Gilligan et al., 2003), resulting in correct expression in hypothalamic OT neurons and furthermore preserved responsiveness to physiological stimuli. Despite the gene conservation, neurons expressing OT-like peptides underwent tremendous evolutionary transformations. Compared to primitive OT neurons contacting the ventricle system or acting in paracrine manner on epithelial cells of the pituitary, OT neurons in advanced vertebrates acquired a voluminous dendritic tree and bifurcating/branching axons supplementary to the preserved early features. The classical neuroendocrine action of systemic release via the posterior pituitary to affect, e.g., the reproductive system and basal reflex-like reproduction was here expanded to influence also cognitive processes in favor of reproduction-related and pro-social behaviors, e.g., to impact partner preference and pair bonding as well as parental care and gregarious socialization. It is difficult to assess when in evolution neurohormonal effects of OT on the reproductive physiology were supplemented by its effects on reproductive behavior. It seems that all vertebrates successfully operate both mechanisms. At least in mammals there is anatomical evidence for dual projection of OT neurons to the systemic release site (the posterior pituitary) and central release sites (the nucleus accumbens and central amygdala) (Ross et al., 2009; Knobloch et al., 2012). Accordingly, synergistic effects of peripherally and centrally released OT were reported for some situations, including stress (Neumann and Landgraf, 2012 and references therein). Such correlation and its functional significance should be further explored in the context of reproduction-related and pro-social behavior, especially in primates.

Going back to the central effects of OT on behavior, it should be noted that in basal vertebrates the behavioral responses are rather slow and stereotypic, therefore, it is likely that they are mostly mediated by trans-ventricular action of OT homologs. In mammals, the evolution established social effects of OT, which exceed classical mating and reproductive behaviors (Figure 5). To exemplify, it was recently reported that central administration of OT in marmoset fathers facilitates food sharing with their infants (Saito and Nakamura, 2011). Such complex and rapidly occurring paternal behavior is likely mediated by targeted OT axonal release (Knobloch et al., 2012) in high brain areas, allowing for modulation of higher order social processing. OT is supposed to preferentially act on interneurons (Knobloch et al., 2012; Owen et al., 2013), which, in turn, relatively rapidly (i.e., within the range of seconds) modify the network activity of certain brain region(s), resulting in fast emotional, behavioral or cognitive responses. Following this idea, the demonstration of universality of the axonal route for central OT release in the context of modulating forebrain activity and elaborate behaviors should be further explored and extended to the advanced placental mammals, namely primates. One day, the stimulation of endogenous OT in the brain might be one approach helping to cure or simply improve the situation of humans afflicted with autism spectrum disorders (Meyer-Lindenberg et al., 2011)—a disease characterized by a deficient social competence on the recognition- as well as the prospecting level, accompanied by reproductive problems reaching up to asexuality (Gilmour et al., 2012).

### Conflict of interest statement

The authors declare that the research was conducted in the absence of any commercial or financial relationships that could be construed as a potential conflict of interest.

## Footnotes

^1^It is important to note that the observation of first gland-like neurons was reported by Speidel (1919; cited from Watts, 2011) in the spinal cord of fish. These cells (known as Dahlgren cells) and their axons form an unique caudal neurosecretory system in fish, terminating in the urophysis and secreting urotensins and CRH to the systemic blood in the same fashion as the release of hypothalamic nonapeptides from the posterior pituitary (McCrohan et al., 2007). For the history of the concept of neurosecretion and the establishment of neuroendocrinology as a new discipline linking neuroscience and endocrinology see the excellent review of Watts (2011).

^2^The AN (in rats) were first described by Peterson (1966). For the anatomy of the AN in representatives of the phylogenetic row and their respective nomenclature see the review of Grinevich and Polenov (1994).

^3^Here we follow the terminology for contacts of magnocellular neurons from Polenov (1978).

^4^The term “neuronalization” was introduced by Andrey L. Polenov about 40 years ago to describe the process of evolutionarily transformation of primitive unipolar neurosecretory cells to typical neurons, which preserved capacity to produce and secret neurohormones (Polenov, 1978). However, presently the term “neuronalization” is used to name the appearance of neuron-like cells from adult liver or bone marrow stem cells (Deng et al., 2006). Therefore—to avoid confusion—we will not further use the term “neuronalization” in the present review.

^5^Early reports (Landgraf et al., 1988; Russel et al., 1992) showed that the local osmotic or naloxone stimulation of the PVN or SON induces an increase in OT concentrations in extrahypothalamic forebrain regions, such as lateral septum, suggesting the central OT release from processes of OT neurons. However, the anatomical and functional evidences for distant axonal OT release were obtained relatively recently (Ross et al., 2009; Knobloch et al., 2012).
